# Association of systemic lupus erythematosus disease activity index score with clinical and laboratory parameters in pediatric onset systemic lupus erythematosus

**DOI:** 10.12669/pjms.36.3.1480

**Published:** 2020

**Authors:** Roshila Shamim, Sumaira Farman, Shabnam Batool, Saira Elaine Anwer Khan, Muhammad Kamil Hussain Raja

**Affiliations:** 1 Roshila Shamim, FCPS (Medicine). Division of Rheumatology, Fatima Memorial Hospital, Lahore, Pakistan; 2 Sumaira Farman, FRCP, FACP, FACR, SCE Rheumatology. Graduate Certificate Paediatric Rheumatology, Dept. of Rheumatology, National Hospital and Medical Centre, Lahore, Pakistan; 3 Shabnam Batool, FCPS (Rheumatology), FCPS (Medicine). Division of Rheumatology, Fatima Memorial Hospital, Lahore, Pakistan; 4 Saira Elaine Anwer Khan, MRCP, SCE Rheumatology. Division of Rheumatology, Fatima Memorial Hospital, Lahore, Pakistan; 5 Muhammad Kamil Hussain Raja, MBBS. Division of Rheumatology, Fatima Memorial Hospital, Lahore, Pakistan

**Keywords:** Clinical and laboratory parameters, Pediatric onset systemic lupus erythematosus (p-SLE), Systemic lupus erythematosus disease activity index (SLEDAI) score

## Abstract

**Objective:**

To determine the association of systemic lupus erythematosus disease activity index (SLEDAI) score in pediatric onset SLE (p-SLE) with clinical and laboratory parameters.

**Methods:**

This cross sectional observational study was conducted at Division of Rheumatology, Fatima Memorial Hospital, Lahore from November 2018 to January 2019. Total 23 patients diagnosed with p-SLE having onset of symptoms at ≤ 18 years of age, irrespective of their current age at presentation, of either gender, fulfilling criteria of 2012 Systemic Lupus International Collaborating Clinics (SLICC) criteria were enrolled. Patients’ clinical symptoms and laboratory parameters were reviewed, SLEDAI scores were calculated. Collected Data were entered in proforma and analyzed on SPSS version 23.

**Results:**

There were 91.3% females. Mean age at diagnosis was 11years ± 4years. At presentation patients had hematological involvement 69.6% followed by mucocutaneous symptoms 65.2% and renal involvement 21.6%. ANA by IFA was positive in all, while anti-ds-DNA was positive in 78.3% patients. SLEDAI score was ≥6 in 87% patients, average SLEDAI score was higher in patients with renal involvement (*p=*0.06). Elevated ESR (r=0.48, *p*=0.02), Anti-dsDNA (r=0.44, *p=*0.05) and low complement levels (*p=*0.03) were significantly positively correlated, while hemoglobin (r= -0.43, *p=*0.04) was negatively correlated with the SLEDAI score.

**Conclusion:**

In this study, patients with Lupus Nephritis had high SLEDAI scores. Elevated Anti-dsDNA titer, ESR, low complement levels and hemoglobin were significantly associated with high SLEDAI scores. We recommend that SLEDAI score should be calculated in p-SLE patients for stringent disease monitoring and treatment.

## INTRODUCTION

Systemic Lupus Erythematosus (SLE) is an autoimmune multi system disease that causes dysregulation of immune system leading to damage of various tissues and organs.^1^ Although it predominantly affects women of childbearing age, it can occur in all age groups, with pediatric onset SLE (p-SLE) comprising 15-20% of all cases.^2^ p-SLE is often a more severe disease with involvement of renal, neurological and hematologic systems^1^ leading to permanent damage when compared to adult-onset SLE patients (a-SLE).^1^ The upper age limit for p-SLE is not clearly defined, with most studies quoting the upper age limit cut off of 16 or 18 years. Data collected from US Medicaid Beneficiary Population for the period 2000-2004, showed an annual incidence of p-SLE of 2.22 per 100,000 children and a prevalence of 9.73 per 100,000 children, between the ages of 3 to 18 years. SLE is more female centric in adult onset SLE (a-SLE), female to male ratio being 9:1 while in p-SLE this ratio decreases to 4-5:1.^2^

As in Rheumatoid arthritis, a treat to target approach is now advocated for SLE.^3^ In order to achieve this it is fundamental to both diagnose, as well as to correctly recognize disease activity or flare. For the diagnosis of SLE, Anti-nuclear antibodies (ANA) by immunofluorescence technique (IFA), with a sensitivity of 90-98%, is considered a gold standard screening test, its specificity is only 78-83%.^4^ Anti-double stranded DNA antibody (Anti-dsDNA) another antibody commonly detected in SLE, it has specificity of 97% but sensitivity of 60%.^5^ By combining these two antibody, we get a higher sensitivity and specificity for the diagnosis of SLE.

For the identification of disease activity there are certain disease measurement tools, among them SLE Disease Activity Index (SLEDAI) is a global score used to assess the severity of disease of the previous ten days, contain both clinical and laboratory parameters.^6^ It is shown to be an excellent tool in assessing the disease activity in p-SLE patients because it is concise and easy to use and has shown remarkable psychometric properties in validation.^7^ Different clinical features like malar rash, oral ulcers and laboratory parameters like hematuria, proteinuria, low complement levels (especially of C3), and an elevated ESR have shown to be associated with increase disease activity.^8^

The aim of this study was to see the association of SLEDAI score with the clinical and laboratory presentation of p-SLE in a Pakistani population presenting to a tertiary care Rheumatology Department. This is a unique study as it is the first to assess disease activity in p-SLE by calculating SLEDAI score and correlates it with clinico-laboratory parameters; all previous studies published locally, and these are scarce, have only highlighted the clinical and laboratory features in p-SLE.^9^,^10^

## METHODS

This cross-sectional study was carried out in the Rheumatology Department of Fatima Memorial Hospital (FMH), Lahore, over a period of three months from November 2018 to January 2019, after approval (Ref. No. FMH-09-2018-IRB-494-M, dated on October 25, 2018) from Institutional Review Board (IRB).

A total of 23 patients diagnosed with p-SLE below or at the age of 18 years, irrespective of their current age, of either gender, fulfilling 2012 criteria of Systemic Lupus International Collaborating Clinics (SLICC) were enrolled, patients with MCTD, overlap syndrome were excluded. No laboratory tests/ intervention has been done for the purpose of this study, it was a standard of care provided to the patients and after informed consent (Appendix I) from patient or parent/ guardian (in case of a minor), their medical records were reviewed for clinical symptoms, and laboratory investigations comprising of erythrocyte sedimentation rate (ESR), hemoglobin level, total leucocyte count (TLC), platelet count, urine complete analysis for proteinuria (by dipstick method), hematuria (> 5 RBCs), pyuria (>5 WBCs) on high power field and spot urine for protein to creatinine ratio; SLE related specific tests, including ANA by IFA, Anti-dsDNA by ELISA technique, complement C3 and C4 levels and histopathology. SLEDAI score (Appendix I) was calculated, patients with SLEDAI score ≥ 6 will be defined as having active disease while those with score < 6 will be considered as having inactive disease.^8^ All these parameters were entered in a pre-designed proforma.

Data were entered and analyzed on SPSS version 23. Mean and standard deviation were calculated for numerical variables while frequencies and percentages were calculated for categorical variables. Non-parametric Spearman correlation and chi-square between SLEDAI score and laboratory investigation were applied to see any association while non-parametric Man Whitney test was applied to see any statistically significant difference between patients having clinical symptoms with high or low SLEDAI score.

## RESULTS

A total of 23 patients were enrolled, in which 91.3% (n=21) were females and 8.7% (n=2) were male, with a female to male ratio of 21:2. The mean age of patients at presentation was 15.4 years ±5, while mean age at diagnosis of SLE was 11 years ±4. Consanguinity between the parents of patients was present in 47.8% (n=11). Mean age of menarche was 12.8±0.8 years. The median of duration of disease, which was ascertained by the onset of symptoms suggestive of SLE to the present time was 36.0 IQR 81.0 months i.e. approximately 3.5 years, with a minimum disease duration of one month to the maximum disease duration of 120 months i.e. 10 years: and most patients i.e. 47.8% (n=11) had disease duration between 1 to 5 years.

At presentation patients had symptoms of fever 43.5% (n=10), mucocutaneous symptoms 65.2% (n=15) that comprised of oral ulcers 26.1% (n=6), malar rash 39.2% (n=9), discoid rash 4.3% (n=1), livedo reticularis 8.7% (n=2), and photosensitivity 30.4% (n=7). Renal involvement was found in 26.1% (n=6) of patients, these patients presented with proteinuria, pyuria and hematuria. On renal biopsy 66.6% (n=4) had WHO class IV diffuse proliferative while 33.3% (n=2) had class V membranous lupus nephritis. Hematological involvement was seen in 69.6% (n=16), patients, as anemia, thrombocytopenia and leucopenia. Serositis was present in 8.7% (n=2) of patients in which one patient presented with pericardial effusion and one with ascites. Neurological involvement was present in 13% (n=3) of patients in the form of seizures (n=1), stroke (n=1) and optic neuritis (n=1). They were diagnose as having NPSLE after complete physical examination as well as laboratory and radiological workup including MRI Brain along with EEG and Optical Coherence Tomography (OCT) in patients with seizures and optic neuritis respectively.

On autoimmune workup ANA by IFA was positive in all patients while Anti-dsDNA was positive in 78.3% (n= 18) of patients. Other laboratory workup showed acute phase reactant ESR was raised in most of the patients. Complements were low in 65% (n=13), normal in 35% (n=7) while not done in three patients. SLEDAI score was ≥6 in 87% (n=20) while less than 6 in 13% (n=3) of patients. For further details. Table-I.

**Table-I:** Frequency of clinical, laboratory parameters and SLEDAI score.

Features	n (%)/23
***Age at diagnosis***	
< 11 years	7 (30.4)
11-14	7 (30.4)
15-18	9 (39.2)
***Gender***	
Female	21 (91.3)
Male	2 (8.7)
***Symptoms/Signs at presentation***	
Fever	10 (43.5)
Mucocutaneous	15 (65.2)
Hair loss	10 (43.5)
Arthritis	10 (43.5)
Renal involvement	6 (26.1)
Hematological	16 (69.6)
Neurological	3 (13.0)
Serositis	2 (8.7)
Raynaud’s	3 (13.0)
***Laboratory parameters***	
Anemia	12 (52.2)
Leucopenia	3 (13.0)
Thrombocytopenia	3 (13.0)
Proteinuria	6 (26.1)
Hematuria	2 (8.7)
Pyuria	6 (26.1)
Raised ESR	18 (78.3)
***Immunological parameters***	
Presence of ANA	23 (100)
Presence of Anti-dsDNA	18 (78.3)
Low complements	13 (65)
***SLEDAI score***	
Active (≥6)	20 (87.0)
Inactive (<6)	3 (13.0)

**Fig.1:**
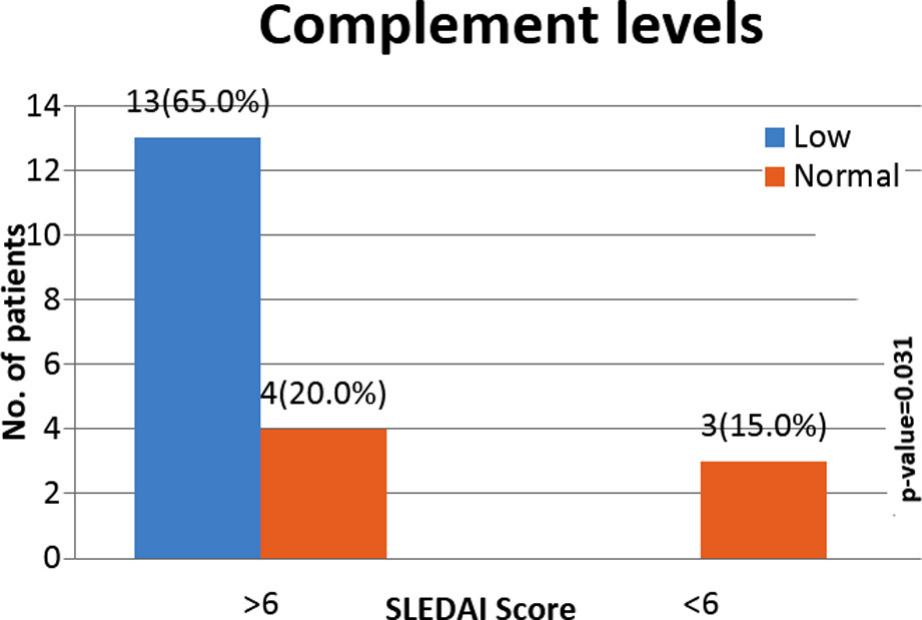
Association of SLEDAI score with complement levels (C3, C4).

In our study we have found significant association between SLEDAI and certain laboratory parameters. It was shown that elevated ESR (r=0.48, *p*=0.02), Anti-dsDNA (r=0.44, *p=*0.05) and low complement levels (*p=*0.03) were significantly positively correlated, while hemoglobin (r= -0.43, *p=*0.04) was significantly negatively correlated with the SLEDAI score, while platelets count value, leucocyte count and protein to creatinine ratio did not show any significant association. Table-II, Fig.1

**Table-II:** Correlation of SLEDAI score with laboratory parameters in p-SLE Patients (n=23).

Laboratory Parameters	Spearman’s correlation with P value Sledai Score (r)
Hemoglobin value	-0.4140.04*
Leucocyte count value	0.3780.07
Platelet count value	0.1520.48
ESR value	0.4590.02*
Protein to creatinine (P.C.) ratio value	0.0290.95
Anti-dsDNA titer value	0.4050.05*

* Correlation is significant at the 0.05 level (2-tailed).

In this study we have found significant differences in SLEDAI scores of different clinical manifestations. Average SLEDAI score was higher in individuals presenting with renal involvement (*p=*0.06) as evidenced by proteinuria, pyuria and hematuria, followed by neurological symptoms and presence of serositis, while patients with mucocutaneous symptoms, arthritis and hematological involvement have less disease activity scores. Table-III.

**Table-III:** Difference between SLEDAI scores of clinical manifestations.

Clinical manifestations	Median SLEDAI score in		p-value

	Presence of manifestations	Absence of manifestations	
Mucocutaneous	10.5	8	0.10
Hair loss	10.5	8	0.15
Arthritis	10.5	8	0.07
Renal	14.5	9	0.06
Hematological	9.5	9	0.83
Neurological	13	8.5	0.21
Serositis	11.5	9	0.34
Raynaud’s	8	9.5	0.28

## DISCUSSION

In this study 21 patients were female with a female to male ratio of 10.5:1, this ratio is higher to the ratio reported in p-SLE i.e. 4-5:1, and closer to the ratio seen in adult SLE i.e. 9:1;^2^ this may be because this study was conducted in a primarily adult rheumatology set up, although in dedicated pediatric clinics, with 21.7% of patients being above 18 years of age. Mean age at diagnosis of disease was 11±4 years, which is similar to another study conducted in Pakistan 9 as well as studies conducted in Asia that reported mean age at diagnosis between 8.6-13.5 years.^11^

Hematological manifestations were the most common manifestations seen in South East Asia region^1^, similarly 69.9% of our patients presented with hematological involvement; Anemia being the commonest manifestation at 52.2%, close to the frequency of anemia seen in p-SLE population in Pakistan^9^ and associated with increased SLEDAI score therefor it is an alert to contemplate flare in p-SLE patients presenting with anemia.

p-SLE is a major cause of mortality and morbidity in the young population^12^, although its survival has been improved in past few years because of early diagnosis and recognition of disease activity^13^, but the survival seen in a Pakistani SLE cohort was less as compared to that of reported Caucasian and Asian series.^14^ Among its clinical symptoms renal involvement is a common feature with a frequency of 37-82%.^15^ The proportion of SLE patients who develop nephritis is higher in Afro-Caribbean’s and Indo-Asians than in whites^16^, contrary in our patient`s cohort renal involvement was slightly less i.e. 26.1%. It has been estimated that proliferative nephritis class IV is the most common histopathology identified in p-SLE and the most aggressive one^1^ likewise 66% of our patients had WHO class IV proliferative nephritis. It has been reported that p-SLE with Lupus Nephritis has high disease activity,^15^ similar results were found in this study, the average SLEDAI score was higher in patients presenting with renal involvement (*p=*0.06) as shown in Table-III. Therefor early diagnosis and effective treatment is necessary in the management of these patients in order to improve long term survival.

Auto immune profile including ANA by IFA was positive in all patients, thus being consistent with its sensitivity of 90-98% in SLE^4^, while Anti-dsDNA was positive in 78.3% of patients, a result similar to other studies conducted in Pakistan and South East Asia.^1^,^8^,^9^

In a-SLE, a rise in Anti-dsDNA titer seen with higher disease activity in most but not in all cases^17^, this principal is also followed in p-SLE^18^, there are studies suggesting a positive correlation between disease activity and titer of Anti-dsDNA.^19^,^20^ Low complement levels are also associated with disease flare.^8^,^20^,^21^ Similar results were found in our patients, increased SLEDAI scores were significantly associated with elevated Anti-dsDNA titer and low complement levels. Inflammatory marker like ESR often increases in SLE patients with active disease,^8^,^22^ likewise in this study, elevated ESR is associated with increased disease activity scores. Above mentioned results suggest that these laboratory parameters are the useful investigation tools for assessing disease activity and predicting SLE flares.

There was no significant association found between any clinical symptoms and SLEDAI score, however, the average SLEDAI score was significantly higher in patients with Lupus nephritis (Table-III).

There is no previous data available on disease activity in p-SLE and its association with clinical and laboratory parameters in Pakistan, this study will guide further research in this field.

### Limitations of the study

It includes the small cohort, conducted at a single center, and a tertiary care referral bias.

## CONCLUSION

Elevated Anti-dsDNA titer, ESR, low complements and hemoglobin were significantly associated with high SLEDAI scores. The average SLEDAI score was significantly higher in our Lupus nephritis patients, and we recommend that all pediatric lupus patients should have a Urine dipstick done routinely in outpatient, and SLEDAI scores calculated to enable stringent disease monitoring and treatment.

### Author’s Contribution:

**RS, SB & SEAK** conceived the study.

**RS** Prepared the manuscript, did data collection, statistical analysis and manuscript writing; & responsible for the accuracy of the work.

**MKHR** did data collection and manuscript writing.

**SF** did review and final approval of manuscript.

## APPENDIX I

**Table:** Systemic Lupus Erythematosus Disease Activity Index (SLEDAI) Score. Check box: If descriptor is present at the time of visit or in the proceeding 10 days.

Wt	Present	Descriptor	Definition
8	□	Seizure	Recent onset. Exclude metabolic, infectious or drug cause
8	□	Psychosis	Altered ability to function in normal activity due to severe disturbance in the perception of reality. Include hallucinations, incoherence, marked loose associations, impoverished thought content, marked illogical thinking, bizarre, disorganized, or catatonic behavior. Excluded uremia and drug causes.
8	□	Organic Brain Syndrome	Altered mental function with impaired orientation, memory or other intelligent function, with rapid onset fluctuating clinical features. Include clouding of consciousness with reduced capacity to focus, and inability to sustain attention to environment, plus at least two of the following: perceptual disturbance, incoherent speech, insomnia or daytime drowsiness, or increased or decreased psychomotor activity. Exclude metabolic, infectious or drug causes.
8	□	Visual Disturbance	Retinal changes of SLE. Include cytoid bodies, retinal hemorrhages, serous exudate or hemorrhages in the choroids, or optic neuritis. Exclude hypertension, infection, or drug causes.
8	□	Cranial Nerve Disorder	New onset of sensory or motor neuropathy involving cranial nerves.
8	□	Lupus Headache	Severe persistent headache: may be migrainous, but must be nonresponsive to narcotic analgesia.
8	□	CVA	New onset of cerebrovascular accident(s). Exclude arteriosclerosis
8	□	Vasculitis	Ulceration, gangrene, tender finger nodules, periungual, infarction, splinter hemorrhages, or biopsy or angiogram proof of vasculitis
4	□	Arthritis	More than 2 joints with pain and signs of inflammation (i.e. tenderness, swelling, or effusion).
4	□	Myositis	Proximal muscle aching/weakness, associated with elevated creatine phosphokinase/adolase or electromyogram changes or a biopsy showing myositis.
4	□	Urinary Casts	Heme-granular or red blood cell casts
4	□	Hematuria	>5 red blood cells/high power field. Exclude stone, infection or other cause.
4	□	Proteinuria	>0.5 gm/24 hours. New onset or recent increase of more than 0.5 gm/24 hours.
4	□	Pyuria	>5 white blood cells/high power field. Exclude infection.
2	□	New Rash	New onset or recurrence of inflammatory type rash.
2	□	Alopecia	New onset or recurrence of abnormal, patchy or diffuse loss of hair.
2	□	Mucosal Ulcers	New onset or recurrence of oral or nasal ulcerations
2	□	Pleurisy	Pleuritic chest pain with pleural rub or effusion, or pleural thickening.
2	□	Pericarditis	Pericardial pain with at least 1 of the following: rub, effusion, or electrocardiogram confirmation.
2	□	Low Complement	Decrease in CH50, C3, or C4 below the lower limit of normal for testing laboratory.
2	□	Increased DNA binding	>25% binding by Farr assay or above normal range for testing laboratory.
1	□	Fever	>38°C. Exclude infectious cause
1	□	Thrombocytopenia	<100,000 platelets/mm3
1	□	Leukopenia	<3,000 White blood cell/mm3. Exclude drug causes.

TOTAL SCORE (Sum of weights next to descriptors marked present).
